# Current Therapeutic Strategies for Stem Cell-Based Cartilage Regeneration

**DOI:** 10.1155/2018/8490489

**Published:** 2018-03-25

**Authors:** Yoojun Nam, Yeri Alice Rim, Jennifer Lee, Ji Hyeon Ju

**Affiliations:** ^1^CiSTEM Laboratory, Catholic iPSC Research Center, College of Medicine, The Catholic University of Korea, Seoul 137-701, Republic of Korea; ^2^Division of Rheumatology, Department of Internal Medicine, Seoul St. Mary's Hospital, Institute of Medical Science, College of Medicine, The Catholic University of Korea, Seoul 137-701, Republic of Korea

## Abstract

The process of cartilage destruction in the diarthrodial joint is progressive and irreversible. This destruction is extremely difficult to manage and frustrates researchers, clinicians, and patients. Patients often take medication to control their pain. Surgery is usually performed when pain becomes uncontrollable or joint function completely fails. There is an unmet clinical need for a regenerative strategy to treat cartilage defect without surgery due to the lack of a suitable regenerative strategy. Clinicians and scientists have tried to address this using stem cells, which have a regenerative potential in various tissues. Cartilage may be an ideal target for stem cell treatment because it has a notoriously poor regenerative potential. In this review, we describe past, present, and future strategies to regenerate cartilage in patients. Specifically, this review compares a surgical regenerative technique (microfracture) and cell therapy, cell therapy with and without a scaffold, and therapy with nonaggregated and aggregated cells. We also review the chondrogenic potential of cells according to their origin, including autologous chondrocytes, mesenchymal stem cells, and induced pluripotent stem cells.

## 1. Introduction

Articular cartilage is a hyaline lining on the articular surface of bone ends. It cushions external impacts and reduces friction between bones to enable smooth and painless joint motion. Chondrocytes are the only resident cell type in cartilage and comprise 1–5% of articular cartilage. These cells produce collagen, proteoglycans, and hyaluronic acid, which are components of the extracellular matrix (ECM) and underlie the mechanical properties of cartilage [1, 2].

Cartilage damage is characterized by gradual destruction of articular cartilage, an avascular connective tissue with a poor regeneration capacity. Damage of articular cartilage results in pain, swelling, and a limited range of motion due to its limited intrinsic healing ability. It can be triggered by pathologic changes caused by trauma, aging, genetic factors, and inflammation. Hypertrophy of chondrocytes and synovial membranes, cartilage degeneration, chronic arthritis, and systemic inflammation can also occur, leading to varying degrees of chondrocytosis, which is the growth of chondrocytes [3].

Several attempts have been made to regenerate articular cartilage. Treatment depends on the condition of the patient and their degree of cartilage damage. In the case of complete cartilage degeneration, total joint replacement is the only option [4]. Microfracture and autologous chondrocyte implantation (ACI) have been proposed as surgical options for partial cartilage lesions. For patients with cartilage degeneration of an intermediate severity, tissue engineering approaches are emerging as a means to restore cartilage more effectively than microfracture or ACI.

Mechanical, biological, and chemical scaffolds can mitigate the disadvantages associated with cell-based therapy, such as insufficient integration into host tissues, inaccurate cell delivery, and degeneration of healthy cartilage. A scaffold-based approach has been developed to better fill cartilage lesions with autologous chondrocytes. When chondrocytes are propagated in a 3D environment, less dedifferentiation occurs and more hyaline cartilage forms [5]. The development of hyaline-like cartilage is improved by implantation of hyaluronic acid scaffolds containing autologous chondrocytes into defect sites [6, 7]. However, despite great efforts to mimic the in vivo environment using biological reactors, exogenous machinery, and biochemical stimulation, tissue with the same properties as healthy cartilage has not been generated [4]. Moreover, the limited number of primary cells (i.e., chondrocytes) reduces the effectiveness of this treatment. Consequently, stem cell-based methods have been developed to avoid the disadvantages associated with primary chondrocyte therapy.

Of the various types of stem cells, bone marrow-derived stem cells (BMSCs) and adipose stem cells (ASCs) have many advantages for clinical applications due to their chondrogenic potential [8–14]. It is easier to separate and proliferate BMSCs and ASCs than primary chondrocytes. These stem cells can differentiate into bone and cartilage and thereby regenerate cartilage in vitro and in vivo [14–19]. However, it is difficult to obtain large numbers of BMSCs and ASCs via in vitro culture because extensive expansion can alter their phenotypes [20–23]. In addition, the yield and differentiation capacity of BMSCs decrease with age and in pathogenic conditions [14, 24, 25]. For these reasons, a new cell source for cartilage regeneration is needed.

In this regard, induced pluripotent stem cells (iPSCs), which can proliferate indefinitely and be produced in large numbers, are of interest. Human iPSCs (hiPSCs) are pluripotent, similar to embryonic stem cells (ESCs), but have no associated ethical problems. hiPSCs can be produced without integrating genes into the genome and can differentiate into chondrocytes in vitro [14, 26]. In addition, a large number of hiPSC libraries prepared from donors, homozygous for the human leukocyte antigen (HLA), have been established. Theoretically, a relatively small number of these HLA-homozygous hiPSC lines would cover the majority of the population.

Here, we summarize the shortcomings and outcomes of various cartilage regeneration strategies and describe various attempts to treat cartilage defects. Moreover, this review discusses stem cell-based engineering to repair cartilage, focusing on hiPSCs. Finally, the future use of hiPSCs for cartilage regeneration is considered.

## 2. Articular Cartilage

Articular cartilage is an elastic connective tissue that covers the ends of bones in diarthrodial joints. It is generated by and composed of chondrocytes. During development, skeletal tissues (including cartilage) are derived from the mesoderm germ layer. Mesenchymal tissues derived from the mesoderm differentiate into chondrocytes. Chondrocytes produce ECM proteins that are rich in proteoglycans. The accumulated ECM proteins lubricate the surface, meaning it can transmit loads without friction [27]. Articular cartilage has a complex composition with various cellular and ECM networks. The characteristics of chondrocytes, the only cell type in articular cartilage, differ according to their location. Chondrocytes located at the surface of articular cartilage produce lubricin, a protein specific to the superficial zone that lubricates the surface [28]. Chondrocytes in the middle zone synthesize a large amount of aggrecan [29]. In deeper regions, most chondrocytes are in a resting state and synthesize proteoglycans. Most synthesized proteins are noncollagenous; therefore, the turnover rate of type II collagen is relatively low. This protein has a half-life of 117 years, unless it is damaged [30].

There are three main types of cartilage: elastic cartilage, fibrocartilage, and hyaline cartilage. Articular cartilage in knee joints is mostly composed of hyaline cartilage. The smoothness and flexibility of hyaline cartilage are intermediate between those of elastic cartilage and fibrocartilage. After a lesion is generated in hyaline cartilage, scar-like tissue (fibrocartilage) forms. It is almost impossible to repair a hyaline cartilage defect by regenerating hyaline cartilage. Moreover, articular cartilage is avascular, alymphatic, and aneural. The lack of blood vessels limits its regeneration ability. The blockade of blood vessels by the dense ECM hampers the delivery of nutrients to damaged cartilage. Chondrocytes receive nutrients by diffusing through the ECM. The low percentage of chondrocytes (1–5%) also hinders the recovery of damaged cartilage [1].

**Figure 1:**
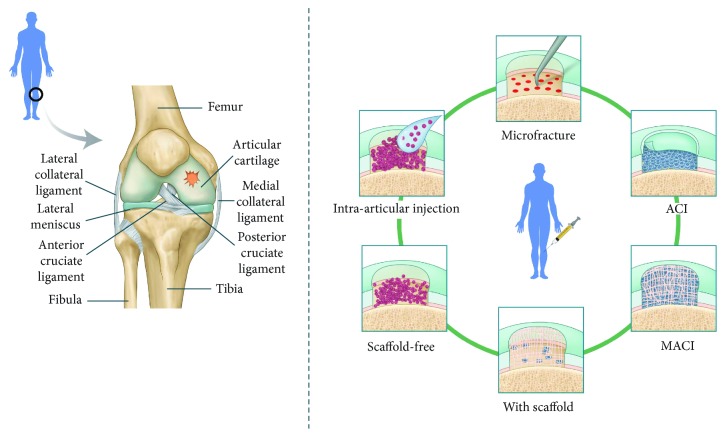
Techniques to regenerate cartilage. Microfracture involves penetrating the osteochondral bone at a depth of 3-4 mm, with each hole separated by 3-4 mm. MSCs migrate from bone marrow to the cartilage defect. ACI involves injecting a patient with their own chondrocytes. MACI involves placing 3D scaffolds, such as those composed of hyaluronic acid or collagen types I and III, into cartilage defects together with autologous chondrocytes. Biocompatible scaffolds have also been developed. There are also scaffold-free techniques that use chondrospheres or self-assembling processes. Smaller chondrospheres are expected to improve therapeutic access via intra-articular injection.

OA, which is related to aging, is the most common form of arthritis and affects millions of people worldwide. Pain is usually caused by the degeneration of articular cartilage in joints [31]. Even the smallest lesion can affect the whole cartilage tissue during the progression of damage. Such cartilage damage is caused by metabolic imbalances [32]. An imbalance between catabolic and anabolic factors leads to cartilage degradation [32]. Pathological changes include cartilage degradation, osteophyte formation, and inflammation. Cartilage degeneration is triggered by sheer stress generated by mechanical forces at the joint surface. This stimulates the proliferation of quiescent chondrocytes and increases their production of ECM proteins and ECM-degrading enzymes [33]. A disintegrin and metalloproteinase with thrombospondin motifs, collagenases, and matrix metalloproteinases degrade collagen II and proteoglycans in the cartilage ECM [2, 34]. This cascade of events degrades hyaline cartilage, which is eventually replaced by fibroblast-like cells. Consequently, hyaline cartilage is replaced by fibrocartilage, leading to stiffness and additional pain. Various strategies have been developed to treat the damaged cartilage and are discussed in this review (Figure 1). A major challenge is to prevent cartilage damage and to regenerate hyaline cartilage.

## 3. Current Repair Approaches for Cartilage Regeneration

### 3.1. Microfracture

Microfracture surgery creates small fractures in the underlying bone. These fractures induce a healing response in damaged articular cartilage by releasing BMSCs [35]. Bone marrow “clots” can firmly adhere to the rough surface of the fractured bone [36, 37]. This promotes the healing of articular cartilage with fibrous tissues or hyaline-like cartilage. Numerous studies report favorable results of microfracture [36, 38–43]. In the late 90s, Bae et al. detected type II collagen at the fracture site in 46 patients with moderate OA at 1 year after surgery [44]. However, studies reported mixed results in the short-, medium-, and long term [45–47]. The quantity and quality of the patient's BMSCs are thought to influence the effectiveness of this approach. Moreover, postoperative rehabilitation is thought to be as important as the surgery itself. Tissue formed following microfracture begins to mature at 8 weeks after surgery [48]. Miller et al. reported that patients who received continuous passive motion (CPM) therapy demonstrated better recovery after microfracture. They concluded that CPM therapy should be performed for 8 weeks after this procedure [49]. Based on this study, CPM for 6–8 hours per day is recommended for patients who have undergone microfracture surgery [50]. However, random cell differentiation induced by microfracture often leads to the formation of fibrocartilage, which is biomechanically inferior to hyaline-like cartilage [44, 51]. Without the mechanical rigidity of hyaline cartilage, the regenerated tissue may deteriorate after 18–24 months and osteophytes may develop due to penetration of the subchondral bone in 25–50% of cases [4, 52]. Moreover, microfracture is less effective for the restoration of large lesions (>3 cm^2^) [53]. Despite these shortcomings, the Food and Drug Administration (FDA) and many clinicians believe that microfracture is a good option for cartilage recovery [54, 55].

### 3.2. ACI and Matrix-Induced ACI

ACI is one of the most promising procedures for long-term cartilage regeneration [53, 56–60]. This method involves obtaining cartilage from the low-weight-bearing part of the joint via a punch biopsy. The isolated cartilage is enzymatically digested to isolate chondrocytes. These chondrocyte are expanded in vitro, transplanted into cartilage defects, and sealed with periosteal flap membranes. Unlike microfracture, ACI is effective for the treatment of large cartilage defects (>3 cm^2^). ACI has yielded favorable clinical and functional results in long-term studies lasting more than 10 years [61–63]. In addition, because this process uses the patient's own cells, potential immune complications are avoided [64, 65]. Matrix-induced autologous chondrocyte implantation (MACI) is an improved version of ACI. Unlike ACI, MACI involves the culture of autologous chondrocytes on type I or type III collagen membranes prior to implantation [66, 67]. This avoids the need to close the defect with watertight sutures [66]. It also helps to maintain the characteristics of articular chondrocytes during long-term cultivation and prevents leakage of chondrocytes inside the joint [68, 69]. However, ACI and MACI both involve two invasive procedures, namely, harvesting chondrocytes and transplanting them back into the patient. Hypertrophy along the flap is also a problem [53]. Alternative membranes, such as porcine membranes composed of a mixture of collagen and hyaluronic acid scaffolds, have been used; however, they can increase the immune response [6, 70, 71]. The effectiveness of these procedures is also limited by the low number of chondrocytes in the harvested cartilage. Indeed, chondrocytes constitute less than 5% of cartilage tissue [60]. Consequently, these cells must be expanded in vitro. However, chondrocytes cultured as a monolayer readily dedifferentiate. Chondrocytes lose their chondrogenic characteristics when grown in a monolayer and start to express fibroblast markers such as collagen type I. Therefore, tissue regenerated using such autologous chondrocytes may be fibrocartilaginous [26, 72].

## 4. Approaches to Improve Chondrogenesis

### 4.1. Scaffolds

Chondrogenesis is thought to require a three-dimensional (3D) environment. During development, chondrogenesis is a complicated process regulated by various growth factors and mechanical factors. A scaffold is commonly used to facilitate in vitro chondrogenesis for tissue engineering [73, 74]. Articular chondrocytes and mesenchymal stem cells (MSCs) are the most commonly used cells in cartilage tissue engineering [75–77]. The structural, mechanical, and biochemical properties of scaffolds can improve cell survival and differentiation. The type of scaffold (i.e., natural or synthetic) is also important [78–81]. Natural biodegradable polymers include polysaccharides, polynucleotides, and proteins, whereas synthetic biodegradable polymers include poly-lactic acid, poly-glycolic acid, and poly-lactic-co-glycolic acid (PLGA) [82]. Scaffolds should ideally be absorptive or biodegradable and support cartilage formation. When creating a scaffold, efforts should be made to ensure it facilitates cell migration. In addition, the pore architecture, elasticity, surface energy parameters, molecular mobility, chemical functionality, pH, and degradation of the scaffold should be considered, as well as any inflammatory responses it may elicit [83]. 3D scaffolds are preferable to two-dimensional (2D) scaffolds for cartilage regeneration. Indeed, 3D structures support cell aggregation, mimic the in vivo environment, and improve cell communication and ECM production [84, 85]. However, scaffolds also have disadvantages. Chondrocyte dedifferentiation, cell death, and cell leakage have been reported in scaffold-based chondrogenesis. Moreover, an inappropriate cell distribution, poor differentiation, and inadequate integration with host tissues are common problems associated with cell transplantation using scaffolds [78, 86, 87]. For example, PLGA scaffolds have been proposed to structurally support cartilage formation [88]. Although these scaffolds yielded promising results for the treatment of full thickness cartilage defects with BMSCs in vivo, their therapeutic efficacy is limited due to the hydrophobicity of PLGA. Efforts are being made to improve cell attachment, function, and differentiation, as well as the scaffold itself [89–91].

### 4.2. Scaffold-Free 3D Culture: Pellet and Micromass Culture

Chondrogenesis is less efficient in a 2D monolayer than in a 3D culture system [92]. Dedifferentiation of chondrocytes in conventional monolayer culture is a major issue for cartilage engineering. In such a system, chondrocytes lose their original characteristics, acquire a fibroblastic morphology, and secrete collagen type I, rather than collagen type II or aggrecan [93, 94]. However, these changes are reversed when dedifferentiated chondrocytes become confluent [95]. Before the emergence of scaffolds, scaffold-free 3D culture systems were generally used for chondrogenesis. These systems mimic precartilage condensation in the developing limb bud and allow cells to interact as they do during cartilage development [96–99]. Cell density significantly influences chondrogenic differentiation [100]. Pellet culture and micromass culture remain the most widely used method for cell-based therapy and cartilage research. Pellet culture of growth plate chondrocytes was first used as an in vitro model of cartilage mineralization [101, 102]. Subsequent studies used this system to study the effects of growth factors on chondrocyte phenotypes, properties of ECM proteins, and the bioenergetics of chondrocytes [103–106]. Early studies of chondrogenesis and hyaline cartilage engineering showed that pellet culture supports in vitro chondrogenesis using MSCs or chondrocytes in the presence of growth factors. Cells differentiated in pellet culture are similar to native articular cartilage in terms of their distribution, density, and matrix composition, without cell phenotypical changes or the assistance of a scaffold [93, 97]. Micromass culture was first performed with chicken limb bud MSCs and was recently used for chondrogenesis. Several studies claim that chondrogenesis is more efficient in micromass culture than in pellet culture. Collagen types I and X are upregulated in larger pellets of chondrogenic cells. Cells trapped in the central region are often undifferentiated and necrotic [107–109]. Although micromass culture supports efficient chondrogenesis, other studies suggest that pellet culture is more suitable for clinical use because of the limited number of chondrocytes generated via micromass culture and their tendency to dedifferentiate [110]. A higher number of cells are required to generate a cartilage-like construct without an artificial scaffold or matrix [111, 112]. Pellet culture and micromass culture are popular methods for in vitro chondrogenesis using various cell types.

## 5. Adult Stem Cell-Based Chondrogenesis

### 5.1. BMSCs

**Figure 2:**
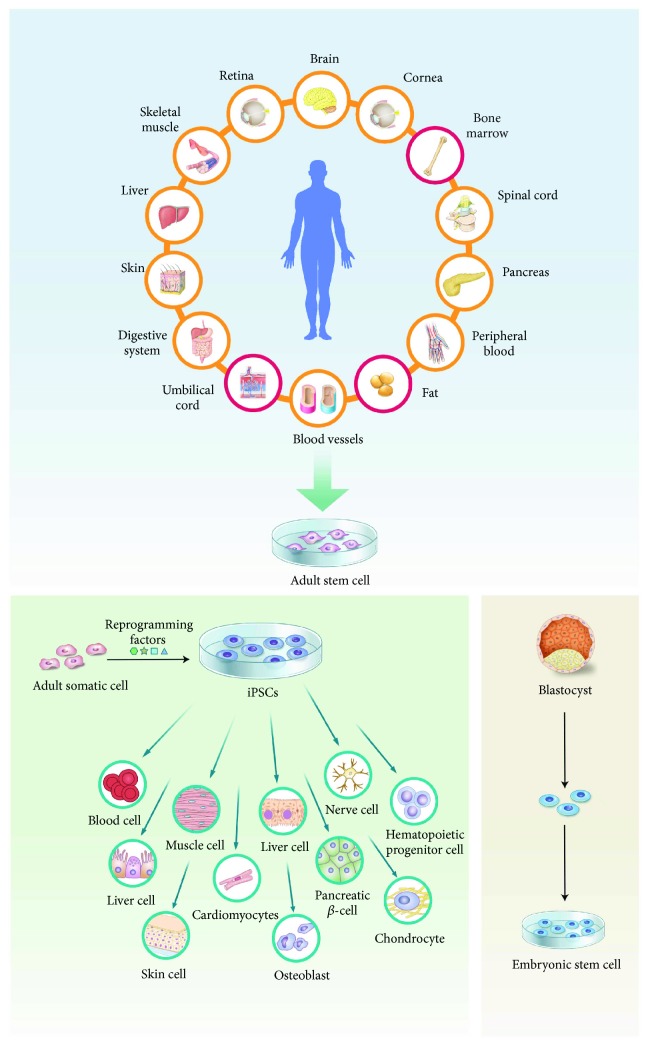
Somatic stem cells (adult stem cells) support healing in the body, for example, replace cells and repair defects. Adult stem cells used in culture are usually obtained from fat, umbilical cord, or bone marrow. ESCs isolated from early embryos can differentiate into various cell types. iPSCs can be artificially generated by reprogramming a patient's own cells and can also differentiate into several lineages.

MSCs can be easily collected from various tissues; however, they are most commonly isolated from bone marrow in humans (Figure 2) [113–115]. Bone marrow stromal cells were first proposed to differentiate into mesenchymal cells, including adipocytes and osteoblasts, in the late 80s via a process called mesengenesis, and these cells were consequently named “BMSCs.” This process has been studied using various in vitro assays. Moreover, BMSCs have been used to treat several diseases [116–121]. In posttraumatic OA models, injection of autologous BMSCs improves the regeneration of joint cartilage exhibiting articular degeneration and osteophyte formation. While autologous chondrocytes are terminally differentiated, BMSCs can differentiate into various cell types (e.g., fibroblasts and chondrocytes) within the joint [122]. The injected BMSCs might also elicit immunomodulatory effects. However, the multipotency of BMSCs is useful for tissue engineering and cartilage regeneration. After several trials with monolayer cultures, aggregates of BMSCs cultured in defined medium were demonstrated to undergo chondrogenic differentiation [25, 97, 123–125]. One important advantage of BMSCs over autologous chondrocytes is that they are more easily expanded in vitro. In vitro chondrogenic differentiation of BMSCs has been widely studied. Treatment with fibroblast growth factor 2 (FGF2) enhances the proliferation and chondrogenic potential of these cells [126, 127]. FGF2-treated BMSCs demonstrate enhanced expansion (increase of 3500-fold versus nontreated BMSCs), increased accumulation of proteoglycans, and downregulation of collagen type I expression. However, BMSCs also have several disadvantages. Patients can experience pain during bone marrow harvesting, and a small volume of bone marrow is obtained, yielding a low number of BMSCs [128]. Only ~1500–3000 fibroblast-forming colonies are obtained from 1 mL of human bone marrow, and it has been suggested that bone marrow biopsies larger than 2 mL are significantly contaminated by peripheral blood [129]. Ex vivo expansion is required to obtain a sufficient number of BMSCs for clinical use, especially in elderly patients and those expected to have few BMSCs [130]. Similar to chondrocytes, most adult stem cells (e.g., BMSCs) exhibit decreased proliferation and a reduced differentiation potential after 4–6 passages [20, 131]. Dexheimer et al. reported that faster proliferation of BMSCs correlates with the formation of larger pellets, fewer apoptotic cells, and higher expression of proteoglycans and collagen type II [132]. Despite the advantages of BMSCs, the slow proliferation rate of cultivated BMSCs and the small number of cells obtained from bone marrow must be resolved.

### 5.2. ASCs

ASCs are obtained by isolating the stromal vascular fraction (SVF) of fat tissue. This is the cell pellet produced when a lipoaspirate, the waste product of liposuction surgery, is digested with enzymes such as collagenase [133]. After serial passaging, adherent cells are harvested as ASCs. Both ASCs and SVFs have a therapeutic potential. The less invasive harvesting procedure and higher yield of ASCs and SVFs have led to these cells being suggested as alternatives to BMSCs. A total of 1 × 10^7^–5 × 10^8^ ASCs are routinely obtained from 300 mL of lipoaspirate, and their viability is >90% [134–137]. This yield is higher than that from bone marrow aspirates, and ASCs are also reportedly easier to culture, proliferate faster, and can be cultivated for longer before becoming senescent [134, 135, 138–140]. Jo et al. investigated the therapeutic effects of intra-articular injection of ASCs for cartilage regeneration in an early phase clinical trial [141]. While ASCs improved cartilage regeneration, numerous studies reported that ASCs can differentiate into cartilage [142–144]. It has been suggested that ASCs have a greater chondrogenic potential than chondrocytes. Indeed, ASCs can maintain their chondrogenic potential for more than 15 passages, longer than chondrocytes [145–147]. However, another study reported that ASCs regenerate cartilage less efficiently than BMSCs [148]. Diekman et al. confirmed that BMSCs expressed a higher level of COL2A1 than ASCs and synthesized more ECM when cultured in alginate beads and a scaffold [149]. Winter et al. showed that ASCs are less sensitive to a chondroinductive environment and that their differentiation is less complete than that of BMSCs after 2 weeks of culture [148]. Chondrogenesis of BMSCs was better than that of ASCs in a 3D system. However, gene expression of aggrecan was higher in ASCs than in BMSCs in the presence of BMP6, while expression of chondrogenic markers was higher in BMSCs than in ASCs in the presence of TGF*β*. Hamid et al. suggested that ASCs should be differentiated prior to passage 4 [150]. Moreover, in that study, expression of chondrogenic markers was highly upregulated at week 1, but decreased at weeks 2 and 3. On the other hand, gene expression of collagen type X was highly unregulated at week 3, indicative of hypertrophy. Chondrogenic induction was only prominent after 1 week of differentiation, even when ASCs were used before passage 4.

### 5.3. Cartilage Regeneration Using Adult Stem Cells

In the surgically induced cartilage damaged animal model, intra-articular injection of labeled BMSCs promoted cartilage tissue regeneration compared to the control group. This result was possible despite the relatively low detection of the labeled BMSCs at the cartilage regeneration site [151]. In addition, when injected with BMSCs in porcine models, cartilage regeneration effect was shown as well [152].

Black et al.'s study assessed the clinical effects of locally derived MSCs in placebo-controlled trials and showed that the range of motion was significantly improved after a single injection of intra-articular adipose-derived MSCs [153].

In mono-iodoacetate-induced rat models, the use of intra-articular BMSCs allowed the animals to distribute significantly greater weight through the affected limb. Despite this functional enhancement, no statistical significant difference appeared between the treatment and the control groups. Also, cartilage and subchondral bone pathology and synovial inflammation were observed in groups treated with BMSC injections [154, 155]. Phases I and II trials using ASCs in the treatment of osteoarthritis (OA) showed MRI evidence of cartilage regrowth [141]. Histological evaluation of collagen type II revealed that hyaline cartilage was regenerated after the injection of 100 million MSCs into a single joint, followed by 6 months of follow-up.

Based on the observed positive preclinical results of using MSCs with arthroscopic techniques, Saw et al. published a randomized controlled trial that included the use of peripheral blood MSCs with arthroscopic microfracture/microdrilling of chondral lesions [156]. As a result, histological analysis and MRI evaluation showed that the quality of cartilage restoration was significantly improved in participants who received MSCs. Another study reported a randomized clinical trial evaluating the efficacy of MSCs after arthroscopic partial medical meniscectomy [157]. The study showed improved clinical outcome compared to the control group and also showed evidences of regeneration of the meniscus volume. Even though preclinical studies have shown inconsistent results, the benefits of intra-articular injection of MSCs or ASCs for improved therapy were proven through various studies.

## 6. iPSCs for Chondrogenesis

### 6.1. iPSCs

hiPSCs were first generated in 2006 by transducing mouse fibroblasts with four Yamanaka factors (Klf4, Oct3/4, c-Myc, and Sox2). In 2007, hiPSCs were successfully produced by introducing KLF4, OCT3/4, SOX2, and c-MYC or SOX2, OCT3/4, NANOG, and LIN28 into human somatic cells [158, 159]. hiPSCs have similar characteristics as human ESCs. However, while hiPSCs can proliferate indefinitely and self-renew, they are not associated with the major ethical issues that complicate the use of ESCs. Therefore, hiPSCs were recently highlighted as an alternative cell source for regenerative medicine [14, 160].

Initially, hiPSCs were routinely generated from skin dermal fibroblasts. However, invasive surgery is required to obtain such cells. Other somatic cells currently used for reprogramming, such as blood cells, urine cells, and keratinocytes, are easier to obtain [161–166]. Cord blood cells, dental pulp stem cells, joint synoviocytes, and adult stem cells (e.g., ASCs and MSCs) have also been successfully reprogrammed [160, 167, 168]. While earlier protocols used lentiviral or retroviral transduction to facilitate integration of Yamanaka factors, hiPSCs should be produced via nonintegrating methods for clinical use. Sendai virus, episomal vectors, small molecules, proteins, and modified RNAs are commonly employed to avoid integration [169–171]. hiPSCs are now widely used for regenerative medicine, drug screening, and even “disease-in-a-dish” modeling.

A pancreas is produced by injecting rat iPSCs into mouse blastocysts that lack a pancreas due to deletion of the Pdx1 gene [172]. Theoretically, human organs can be generated by injecting hiPSCs into pig blastocysts; however, this is complicated by technical issues [26, 173]. Moreover, the blood vessels of iPSC-derived organs are formed by the host animal [26]. This is not a problem for the generation of cartilage because this tissue is avascular [26]. Reprogrammed iPSCs exhibit pluripotency when transplanted into immunodeficient mice, and they generate teratomas containing tissues of all three germ layers (endoderm, mesoderm, and ectoderm). Cartilage-like tissue is found in these teratomas, demonstrating that hiPSCs can undergo chondrogenesis. Therefore, hiPSCs are a promising cell source for chondrogenic tissue engineering.

### 6.2. Cartilage Regeneration Using hiPSCs

The ultimate goal of regenerative medicine with iPSCs is not only to produce cells of interest but also to create new tissues or organs [26, 172, 173]. Teratomas generated from hiPSCs contain hyaline cartilage. This indicates that hiPSCs can differentiate into human cartilage or chondrocytes [26]. Before the emergence of hiPSCs, attempts were made to induce chondrogenesis of human ESCs via three methods: coculture with articular chondrocytes, induction of MSC-like cells, and direct conversion [26, 174–182].

**Figure 3:**
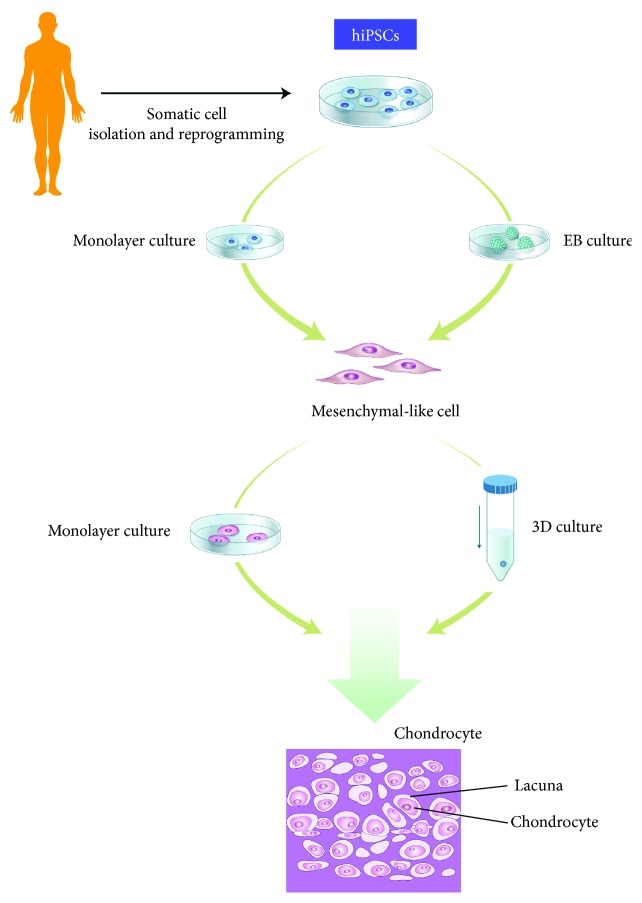
A simple scheme of the various methods used to differentiate iPSCs into chondrocytes.

Protocols used to induce chondrogenic differentiation of hiPSCs were mostly derived from these methods (Table 1). The most widely used approach induces MSC-like cells from hiPSCs (Figure 3). There are several protocols to obtain these progenitor cells, which can be roughly classified into two types: (1) monolayer culture and (2) embryoid body (EB) formation. The latter is more commonly used because it involves mesodermal induction via a defined process. In an early study, Medvedev et al. attempted to induce chondrogenesis of hiPSCs derived from fetal neural stem cells isolated from human embryos [183]. Chondrocytes generated from these hiPSCs exhibited characteristics of chondrocytes. EBs were generated from these hiPSCs, and the media was replaced by chondrogenic differentiation medium. Expression of ECM proteins such as collagen type II and the early chondrogenic marker Sox9 was subsequently detected. In 2012, Zhu et al. also induced chondrogenic differentiation of EBs. Koyama et al. suggested a more defined protocol for in vitro chondrogenesis of hiPSCs in 2012 [184]. While EBs are usually used for differentiation into the three germ layer lineages, they suggested a new protocol for differentiation into the mesenchymal progenitor cell lineage. EBs were generated and grown on gelatin-coated plates for 1 week, after which mesenchymal-like cells or “outgrowth cells” sprouted from these EBs. The outgrowth cells had similar characteristics as MSCs, such as expression of CD44, CD90, CD73, and CD105. The authors reported that 1-2 × 10^4^ cells/cm^2^ was the ideal density for proliferation. Outgrowth cells were dissociated, cell clumps were removed with a strainer, and chondrogenic differentiation was induced. Chondrogenic pellets generated from hiPSC-derived mesenchymal progenitor cells had a cartilage morphology and contained lacuna. Both hiPSCs and human ESCs were successfully differentiated toward the chondrogenic lineage using this protocol. Our group also confirmed the successful chondrogenic differentiation of cord blood-derived hiPSCs via this method [14, 185]. In 2012, Diekman et al. suggested a different approach for chondrogenesis [186]. They isolated cells that expressed collagen type II tagged with green fluorescent protein (GFP) and induced chondrogenesis. Similar to the study by Koyama et al., chondrogenic differentiation was not directly induced. hiPSCs were first predifferentiated into the chondrogenic lineage by micromass culture, and then these cells were dissociated and aggregated by pellet culture. Pellets of GFP+ cells were larger and produced more glycosaminoglycan ECM proteins than those of GFP− cells. The authors concluded that this protocol enhances the chondrogenic properties of engineered tissue and could be a way to eliminate undifferentiated cells, which may prove helpful for transplantation of hiPSCs in the future. However, protocols using EBs are time-consuming and are thought to give rise to a heterogeneous population due to variations in cell number and EB size [175]. Several researchers attempted to differentiate hiPSCs directly without EBs via micromass or pellet culture using specific medium, a coated matrix, or feeder layers [187–189]. However, these methods negatively affect related signaling pathways. Therefore, a fast, inexpensive, and efficient protocol is required for future applications.

### 6.3. Cartilage Regeneration Using hiPSCs in Animal Models of OA

The healing ability of chondrogenic cells derived from hiPSCs was recently investigated in several animal models. In 2014, Ko et al. induced chondrogenic differentiation via EB culture and using alginate beads [16]. Cells in EBs were dissociated and transferred to chondrogenic differentiation medium for micromass culture. The generated chondrogenic pellets or alginate hydrogels were implanted into osteochondral defects created on the patellar groove of immunodeficient rats. Twelve weeks later, the defects were filled with smooth and firm tissue, while the control group had a rough surface with or without fibrous tissue. Histological analysis also demonstrated the restoration of proteoglycans in the defected areas. However, the authors reported that matrix formation was inadequate due to the implantation of allogenic iPSCs, despite the persistence of implanted hiPSCs and the use of immunodeficient rats.

In 2015, Nejadnik et al. generated chondrogenic pellets of ASC-derived hiPSCs [131]. They induced differentiation into the mesenchymal lineage via monolayer culture because they felt that EB culture gave inconsistent results. To confirm the quality of the iPSC-derived mesenchymal cells, hiPSC-derived MSCs and hiPSC-derived chondrogenic cells were differentiated for 21 days and seeded into a polyethylene glycol and chondroitin sulfate methacrylate-based scaffold. Pellets were implanted into an osteochondral defect generated in the distal femur of nude rats and evaluated via serial imaging for 6 weeks. Magnetic resonance imaging showed that the implants did not form teratomas and also detected a decreased water content and increased ECM formation, indicative of successful engraftment. Moreover, the cells expanded in vivo and the scaffold was eventually degraded. Histology confirmed the engraftment of both hiPSC-derived MSCs and chondrogenic pellets in the defect, as demonstrated by Alcian blue and collagen type II staining. Chondrogenic pellets expressed higher levels of matrix proteins; however, hiPSC-derived MSCs also promoted regeneration.

Chondrocytes derived from hiPSCs were implanted into a nonsurgical monosodium iodoacetate- (MIA-) induced cartilage damaged rat model. Zhu et al. generated chondrogenic pellets via EB culture and induction of outgrowth cells [190]. Rather than pelleting the outgrowth cells, they cultured them in chondrogenic differentiation media. Under these conditions, the sprouting outgrowth cells were thought to be chondrocytes. Thereafter, the authors injected 500 *μ*L of the cell suspension (1 × 10^6^ cells/mL) into the joint at 1 week after induction of damage using MIA. Fifteen weeks later, rat knee joints were imaged by microcomputed tomography and analyzed by histology. hiPSC-derived chondrocytes had a better regeneration capacity than hiPSCs. Histological analysis demonstrated that the injected hiPSC-derived chondrocytes localized on the cartilage surface and increased the level of proteoglycans. Proliferating chondrocytes were also detected, suggesting that cartilage was being repaired. The authors concluded that joint function was improved; however, rat joints still showed dyskinesia and did not fully repaired, indicating that stem cell injection can improve joint repair, but only in early stage of destruction.

In 2015, Yamashita et al. implanted hiPSC-derived chondrogenic cells into larger animals. Cartilaginous nodules were generated from a monolayer of hiPSCs [191]. These nodules eventually separated from the bottom of the dish and were transferred to a petri dish and maintained in suspension culture for up to 42 days. The nodules did not form tumors when subcutaneously implanted into severe combined immunodeficiency (SCID) mice. Cartilaginous particles maintained for 28 days were implanted into osteochondral defects of SCID rats. Repair of the defects was confirmed at 4 weeks after transplantation, with high expression of collagen type II. Expression of ECM proteins was higher in nodules maintained for 42 days than in those maintained for 28 days. To determine if human cells migrated to other organs or lymph nodes, the authors investigated expression of human *β*-actin. This was not detected in any other organ, and pluripotency markers were not expressed in nodules differentiated for 21 days or longer. Finally, the nodules were implanted into cartilage defects of mini-pigs weighing 27.0–30.5 kg and treated with the immunosuppressant cyclosporine. The nodules were viable for 1 month after transplantation. Chondrogenic nodules expressed human vimentin and integrated with host articular cartilage. The authors concluded that the nodules can repair cartilage defects even under heavy-weight-bearing conditions.

Taken together, these results demonstrate the potential of hiPSC-derived cartilaginous particles for articular cartilage regeneration. Early phase animal trials are being conducted, the results of which will help to translate this procedure into future clinical applications.

## 7. Conclusions and Future Perspectives

Cartilage damage causes joint destruction, pain, physical disability, and morbidity. However, the avascular nature and low mitotic activity of cartilage limit its intrinsic regeneration capacity. Although biological agents may slow cartilage degradation, the optimal treatment to promote cartilage repair has not been defined.

Cell-based therapies are emerging as a means to regenerate cartilage. One of the points to consider is the stability of the cells before use. Rubio and colleagues questioned the safety of locally derived MSCs through a controversial study in 2005 [192]. When BMSCs were implanted in immunodeficient mice, spontaneous stem cell mutation and malignant tumors appeared. Later, the study was withdrawn after evidence showed that the malignant traits were associated with contamination of cell lines, but not with MSCs [192]. In a similar situation, further studies on long-term cultured BMSCs (evidence of malignant transformation) were withdrawn on the same grounds [193, 194]. Importantly, current clinical trials have shown that MSC therapy is safe. Safety has been demonstrated through a recent systematic review and meta-analysis of a total of 1012 participants who received intravascular MSC therapy for a variety of clinical symptoms, including ischemic stroke, Crohn's disease, cardiomyopathy, and ischemic heart disease [155, 195]. In the case of hiPSCs, the risk of teratoma formation is the biggest problem that cannot be overlooked. Therefore, efforts (i.e., complete differentiation or purification) to avoid this risk are necessary.

Autologous chondrocytes and adult stem cells (i.e., BMSCs and ASCs) are generally used for cartilage regeneration; however, the low numbers of these cells limit their clinical applications. By contrast, hiPSCs can proliferate indefinitely and support chondrogenesis in vitro and in vivo. Using a defined quality control process and a chondrogenic differentiation protocol, hiPSCs can become the ideal cell source for cartilage engineering.

**Figure 4:**
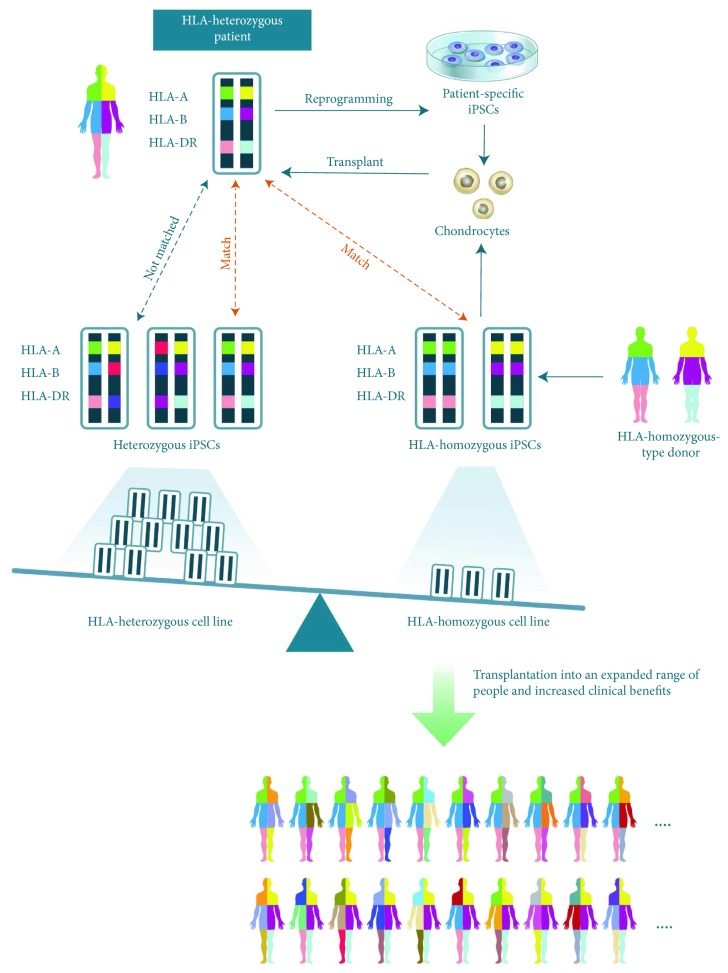
The clinical transplantation of hiPSCs homozygous for HLA-A, HLA-B, and HLA-DR, the three types closely related to immune rejection. Theoretically, a relatively small number of homozygous stem cell lines would cover the majority of the population.

hiPSCs have several advantages. These cells can be theoretically generated from every individual; however, this is not economically viable. It is expensive to prepare hiPSCs from a patient under good manufacturing practice (GMP) guidelines [196]. Consequently, the concept of a HLA-homozygous hiPSC bank has emerged [197]. It is estimated that 100 HLA-homozygous hiPSC lines from each race would cover the majority of the population [198]. Cartilage is considered to be an immunoprivileged tissue due to its avascular and alymphatic nature and the dense ECM that surrounds chondrocytes [26]. The use of HLA-matched hiPSCs may minimize immune rejection during allogeneic transplantation for cartilage engineering (Figure 4). HLA-A, HLA-B, and HLA-DR are closely related to rejection [199, 200]. Many researchers are currently collecting cells homozygous for these three HLA types; however, further research is required to improve allogeneic transplantation of neocartilage.

**Figure 5:**
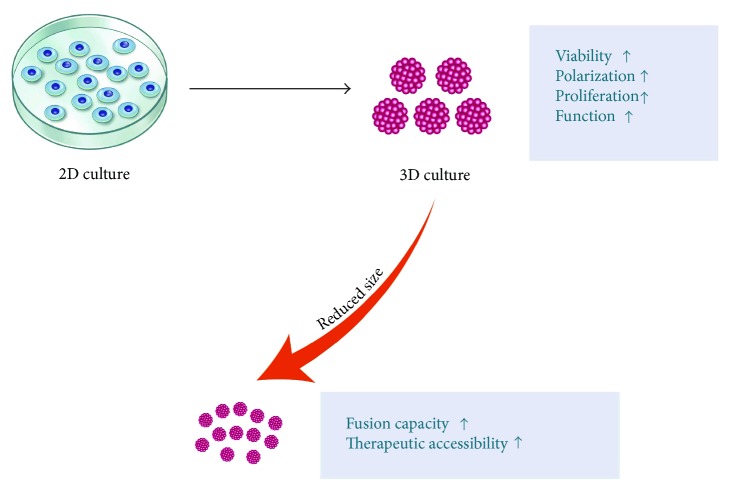
A 3D culture method for tissue engineering. Cells cultured in a 3D system have considerably improved biological properties and a higher regeneration potential than cells cultured in a 2D system. Sophisticated techniques for mass production of spheroids are also being developed. “Micropellet” 3D culture may also improve therapeutic accessibility by reducing the size of the product.

Many cartilage transplantation procedures currently involve surgery. Further damage to the knee joint might exacerbate the immune reaction and hamper recovery. Therefore, the development of an accessible and noninvasive treatment might be ideal for cartilage recovery. Various treatments involve a single injection of cells (e.g., Cartistem). Intra-articular injection of BMSCs and ASCs has been investigated as a means to treat OA [201–208]. Zhu et al. demonstrated that hiPSC-derived chondrocytes may also be clinically applicable by noninvasive procedures. However, chondrocytes demonstrate better viability and function in 3D conditions. Moreover, the properties of cells are better in spheroids than in 2D systems [209]. Specifically, cells in spheroids have a higher viability and readily polarize [210]. Furthermore, the use of scaffold-free spheroids avoids the biocompatibility issues associated with the implantation of scaffolds. This approach can improve tissue regeneration in clinical settings. Spheroids can spontaneously fuse with each other and thereby increase in size [209]. Researchers currently pellet 1–5 × 10^5^ cells in the presence of growth factors [98, 110, 211–214]. Babur et al. termed such aggregates “macropellets” and reported that large spheroids (1-2 mm) are characterized by redifferentiation, with varying amounts of ECM deposited throughout the pellet [215]. Using a microwell technique, they generated “micropellets” measuring 193 ± 20 *μ*m. These small pellets produced higher levels of ECM proteins, which is thought to be related to the increased contact of cells with the surrounding environment. Taken together, we believe that intra-articular injection of a minimized chondrogenic pellet or spheroid is ideal to regenerate damaged cartilage (Figure 5).

**Figure 6:**
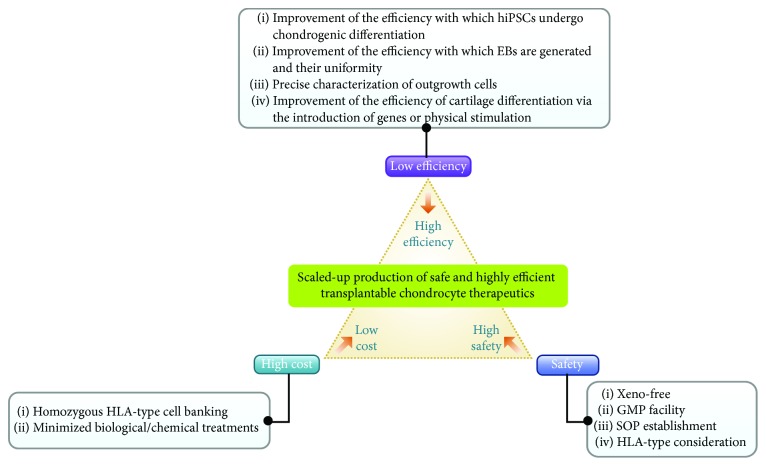
Commercialization strategy to develop safer, more efficient, and less expensive therapeutic agents for cartilage repair using iPSCs.

The potential of hiPSCs to regenerate cartilage has not been investigated in a preclinical or clinical study [216]. However, the use of hiPSCs in cartilage research may promote their applications in other fields, including tissue engineering, drug screening, and modeling of various diseases related to cartilage or even bone. With more defined protocols (e.g., uniform EB generation, defined production of outgrowth cells, and enhanced chondrogenic differentiation), it may be possible to generate spheroids of hiPSCs that readily undergo chondrogenic differentiation (Figure 6). Furthermore, the use of xeno-free components, GMP practices, and other quality control methods, such as the removal of tumorigenic cells, is required for clinical use. These process may allow the production of animal component-free cells with low tumorigenecity from hiPSCs. The high cost of tissue engineering can also be reduced by using homozygous-HLA hiPSCs which requires minimal biological and chemical treatments. In summary, hiPSCs may open up a new era in cartilage regeneration.

## Figures and Tables

**Table 1:** Studies that attempted to induce chondrogenic differentiation of iPSCs.

Year	Reference	MSC-like cell induction method	Chondrogenesis method	Primary cells used for reprogramming	Growth factors	Animal experiments
2011	Medvedev et al. [183]	—	Direct media change of EBs	Human fetal neural stem cells	TGF*β*3, BMP2	—
2012	Koyama et al. [184]	EB formation, outgrowth cell induction	Pellet culture	Human dermal fibroblasts	TGF*β*3	—
2012	Diekman et al. [186]	Micromass culture for predifferentiation	Pellet culture	Mouse fibroblasts	TGF*β*3	—
2013	Guzzo et al. [216]	Monolayer culture on gelatin-coated plates	Micromass culture, pellet culture	Human dermal fibroblasts	BMP2	—
2014	Guzzo et al. [216]	—	Micromass culture	Human dermal fibroblasts, human articular chondrocytes, human cord blood cells	BMP2	—
2014	Ko et al. [16]	EB culture	Micromass culture	Human dermal fibroblasts	TGF*β*3	hiPSC-derived chondrocytes implanted into osteochondral defects of immunosuppressed rats as pellets or in alginate hydrogels
2015	Nejadnik et al. [131]	Monolayer culture	Pellet culture	Human adipose-derived stem cells, human dermal fibroblasts	TGF*β*3	hiPSC-derived chondrocytes expressed human vimentin and integrated with host articular cartilage
2015	Yamashita et al. [191]	Monolayer culture	Monolayer culture to induce cartilaginous nodules	Human dermal fibroblasts		Chondrogenic nodules implanted into defected regions of SCID rats and mini-pigs
2016	Zhu et al. [190]	—	Direct media change of EBs and transfer to gelatin-coated plates for chondrocyte induction	Human dermal fibroblasts	TGF*β*1	Single hiPSC-derived chondrocytes directly injected into the knee joints of a MIA-induced rat model of OA
2017	Nam et al. [14, 185]	EB formation, outgrowth cell induction	Pellet culture	Human cord blood mononuclear cells	TGF*β*3, BMP2	—
